# Ward-Based Small-Group Teaching to Improve Nursing Staff Training Coverage, Confidence, and Self-Reported Practice in Inpatient Falls Prevention: A Before-and-After Quality Improvement Study

**DOI:** 10.7759/cureus.119360

**Published:** 2026-11-09

**Authors:** Meet P Khistariya, Sakshi Watarkar, Ramanathan Annamalai, Mohammed Nayeem, Anshoo Yadav, Simran Kaplash, Biran Antony, Arvin Raju

**Affiliations:** 1 Acute Medicine, University Hospitals of Leicester NHS Trust, Leicester, GBR; 2 General Medicine, University Hospitals of Leicester NHS Trust, Leicester, GBR; 3 Trauma and Orthopaedics, University Hospitals of Leicester NHS Trust, Leicester, GBR

**Keywords:** before-and-after study, falls prevention, falls risk assessment, inpatient falls, in-service training, nursing education, patient safety, quality improvement

## Abstract

Introduction

Inpatient falls are among the most common patient safety incidents in acute hospitals, contributing to patient harm, prolonged hospital admissions, and increased healthcare costs. Adherence to the University Hospitals of Leicester (UHL) Falls Prevention Policy is essential to minimise avoidable falls. However, a baseline staff survey conducted on a general medical ward at Leicester Royal Infirmary highlighted gaps in nursing staff training, familiarity with the policy, confidence in understanding its requirements, and self-reported adherence. This study aimed to evaluate whether targeted ward-based teaching was associated with changes in nursing staff training coverage, familiarity with the policy, confidence in understanding it, and self-reported falls prevention practices.

Methods

A prospective, single-centre, before-and-after quality improvement study was conducted between February 2024 and October 2024. A structured questionnaire, developed based on the UHL policy, assessed staff knowledge and self-reported practices relating to falls risk assessment, enhanced falls reduction measures, documentation, communication and post-fall management. The questionnaire was completed by 32 nursing staff before the intervention (February 2024) and 22 following the intervention (October 2024). The intervention involved presenting baseline findings to the ward team, followed by 60-minute teaching sessions delivered every four weeks throughout the study period. Sessions were conducted during routine shifts in groups of three to four staff members. All outcomes were self-reported, and proportions were compared using Fisher’s exact test.

Results

The proportion of staff reporting falls prevention training within the previous year increased from 21.9% to 90.9% (p < 0.001), while the proportion reporting routine falls risk assessment increased from 71.9% to 95.5% (p = 0.036). The proportion of staff reporting the highest level of confidence in their understanding of the policy also increased substantially, from 40.6% to 100.0% (p < 0.001). Prompt reporting of falls (87.5% to 95.5%, p = 0.638) and documentation of assessments and interventions (93.8% to 95.5%, p = 1.000) were already high at baseline and showed no statistically significant change. Staff familiarity with the policy and reported encouragement of mobility aid use both increased significantly, from 65.6% to 95.5% (p = 0.017).

Conclusions

A low-cost programme of repeated, ward-based small-group teaching was associated with improvements in training coverage, familiarity with the policy, staff confidence, and self-reported falls risk assessment practices. The programme was also feasible to implement within the demands of a busy medical ward. However, as knowledge and clinical practice were not assessed objectively, these findings require further validation using objective measures. Future work should also evaluate whether the observed improvements translate into a reduction in inpatient falls.

## Introduction

Falls are among the most frequently reported patient safety incidents in acute hospitals and are associated with physical injury, reduced confidence and independence, prolonged hospital stays, and increased healthcare costs [1-3]. Reported fall rates in acute care settings range from 0.4 to nine falls per 1,000 patient days [2]. Furthermore, a multi-hospital study conducted in the United States reported that the average total cost of an inpatient fall exceeded US$60,000 [3]. Falls rates vary across clinical specialties, with medical wards accounting for a substantial proportion of this burden. Patients admitted to these wards are often older, acutely unwell and affected by delirium, polypharmacy and reduced mobility [2]. Consequently, researchers have recommended prioritising educational interventions for healthcare professionals working in specialties with higher falls rates [2].

International and national guidelines recommend that older inpatients, as well as younger inpatients at increased risk, undergo a multifactorial assessment to identify individual falls risk factors, followed by tailored interventions rather than relying on a single risk-prediction score [1,4]. In the UK, the National Institute for Health and Care Excellence (NICE) updated its falls guidance in 2025, expanding its scope to include people in hospital and recommending that falls risk-prediction tools should not be used in any setting [4]. Among the interventions evaluated in hospital settings, education has the strongest supporting evidence. A systematic review and meta-analysis of hospital falls prevention interventions found that education targeting both patients and healthcare staff was the only intervention associated with statistically significant reductions in both falls rates and the odds of falling [5].

However, translating falls prevention policies into consistent bedside practice remains challenging. A 2025 systematic review of 55 studies found that the implementation of falls prevention guidelines across healthcare services was generally poor. Staff training and education were the most commonly used implementation strategies. Although the adoption of recommended practices by healthcare professionals frequently improved, almost half of the studies assessing the sustainability of these changes reported a deterioration in practice after six months [6]. Similarly, the implementation of the 2022 World Falls Guidelines has been inconsistent across countries [7]. Qualitative studies involving hospital clinicians have highlighted the influence of workload, competing clinical priorities and the need for shared responsibility among ward teams on the delivery of falls prevention education in practice [8,9]. Therefore, educational approaches that can be integrated into routine ward activities, without requiring staff to be released from their clinical duties, may offer a more practical and achievable approach to falls prevention education in busy acute ward settings.

At the University Hospitals of Leicester (UHL) NHS Trust, the Falls Prevention Policy outlines requirements for falls risk assessment, enhanced falls reduction measures for patients at high risk, documentation, communication and post-fall management, with a departmental target of 100% staff knowledge and compliance. However, a baseline staff survey conducted on a general medical ward at Leicester Royal Infirmary identified gaps in nursing staff training, confidence and self-reported adherence to the policy. This study aimed to evaluate whether a targeted, ward-based educational intervention was associated with changes in nursing staff training coverage, familiarity with the UHL Falls Prevention Policy, confidence in understanding its requirements, and self-reported falls prevention practices. As the study focused on ward nursing staff rather than patients, patient-level characteristics, including age, sex, and frailty, were outside the scope of the study objectives.

## Materials and methods

Study design and setting

This was a prospective, single-centre, before-and-after quality improvement study in which the same anonymous staff questionnaire was administered before and after an educational intervention. It was conducted on a 50-bed general medical ward at Leicester Royal Infirmary, which is part of UHL NHS Trust, a large teaching hospital trust in the East Midlands of England. The ward provides care for adult medical patients, many of whom are older and at increased risk of falls. The study was conducted from February 2024 to October 2024. The pre-intervention survey, which served as the baseline assessment, was conducted in February 2024, followed by the delivery of the educational intervention from March 2024 to September 2024. The post-intervention survey was conducted in October 2024.

Policy standard

The standard used to assess self-reported practice was based on the version of the UHL Falls Prevention Policy in force during the study period, with a departmental target of 100% of nursing staff demonstrating knowledge of and reporting adherence to its key requirements. These requirements included completing falls risk assessments at specified points in the patient journey, implementing enhanced falls reduction measures for patients identified as being at high risk, undertaking bedrail risk assessments, encouraging the use of appropriate mobility aids, timely documentation of assessments and interventions, communicating falls risk between staff members, and the prompt reporting and management of falls.

Participants

All nursing staff working on the ward during each survey period were eligible to participate, including full-time nursing staff, agency or bank nursing staff, and healthcare support workers. Participation was voluntary, and all questionnaires were completed anonymously. A total of 32 staff members completed the questionnaire before the intervention, compared with 22 after the intervention. No formal sampling method was employed; instead, all eligible staff members present during each survey period were invited to participate, resulting in self-selected respondents in both phases. As responses were anonymous, individual participants could not be identified or their responses linked across the two phases. Consequently, the pre- and post-intervention responses were analysed as independent samples.

Questionnaire

A structured questionnaire was developed by the study team based on the content of the UHL Falls Prevention Policy and administered electronically using Google Forms (Google LLC, Mountain View, CA). It comprised 21 questions, numbered consecutively from 1 to 21, organised into five sections. The first section recorded the respondent’s professional role and years of experience. The knowledge and awareness section assessed familiarity with the policy, falls prevention training within the previous year, the most common risk factor for falls, the key principles of falls safety, and the criteria for initiating enhanced falls reduction measures. The implementation section covered the frequency and timing of falls risk assessments, documentation, interventions used for high-risk patients, the level of supervision required following a fall, the timing of falls risk communication, and prompt reporting of falls. The equipment and environment section addressed the encouragement of assistive devices, assessment of baseline mobility and equipment requirements, and measures to reduce falls risk. The final section covered feedback systems, methods of keeping up to date with the policy, overall confidence in understanding the policy, and a free-text question for additional comments. With the exception of questions 5-7, which directly assessed knowledge, the questions required staff to report their own familiarity, training, confidence, and clinical practices.

Questions were answered using single-choice and multiple-response formats, a free-text question, and 5-point rating scales, ranging from never to always or, for confidence, from low to high. For analysis, responses to yes/no questions were reported as the proportion of respondents answering yes. For the rating scales, only a score of 5 was classified as indicating routine or prompt practice, while a score of 5 on the confidence scale represented the highest level of confidence. For the two multiple-response questions included in the analysis, a response was classified as positive only when all policy-required options were selected. For question 9, these included falls risk assessment on admission, following a fall, and on transfer to a new ward. For question 11, these comprised the core nursing assessments of mobility and elimination, together with the adult bedrail risk assessment. The same questionnaire was administered before and after the intervention, and the full questionnaire is provided in the Appendix.

Educational intervention

Following the pre-intervention survey, the findings were presented to the ward team. A programme of ward-based teaching sessions was subsequently delivered to small groups of three to four staff members during routine clinical shifts. Each session lasted 60 minutes and was delivered by members of the study team. Sessions were conducted every four weeks between March 2024 and September 2024, with each teaching round repeated on different days and shifts to maximise staff participation, including that of night-shift and part-time staff. The teaching content was tailored to address the gaps identified in the pre-intervention survey and covered the appropriate timing of falls risk assessments, including on admission, following a fall, and on transfer to a new ward; enhanced falls reduction measures for high-risk patients; core nursing and bedrail risk assessments; the use of appropriate mobility aids; documentation requirements; communication of falls risk during handovers; and post-fall management and incident reporting. The sessions were interactive, allowing staff to raise questions, discuss concerns, and provide suggestions regarding falls prevention practices on the ward. Attendance was recorded using a signed attendance sheet at each session. In total, eight sessions were held, and all nursing staff on the ward attended at least one session.

This format was chosen to maximise staff participation while minimising disruption to patient care, as it did not require staff to be released from their clinical duties and involved no additional funding or equipment. However, the impact of the teaching sessions on ward workload was not formally assessed.

Outcome measures

The primary outcomes were the proportions of staff reporting falls prevention training within the previous 12 months, routine completion of falls risk assessment, consistent documentation of falls risk assessments and interventions, prompt reporting of falls, and the highest level of confidence in understanding the policy. Secondary outcomes were responses to questions on familiarity with the policy, the timing of risk assessment, core nursing and bedrail risk assessments, and the use of mobility aids. Questions one and two recorded respondent characteristics. Responses to questions 5-7, 12, 13, and 16-19 from the pre-intervention survey were used to identify gaps and to plan the content of the teaching; these questions were not prespecified as outcomes and were not included in the comparative analysis. The free-text comments (question 21) were not analysed.

Statistical analysis

Responses were summarised as numbers and percentages. Proportions before and after the intervention were compared using the two-tailed Fisher’s exact test, which was chosen because of the small sample sizes and low expected cell counts. A p-value below 0.05 was considered statistically significant. No adjustment was made for multiple comparisons, and the analysis was considered exploratory. Analyses were performed using Python version 3.12 (Python Software Foundation, Wilmington, DE) with the SciPy library, version 1.17.

Ethical considerations

This study involved human participants: nursing staff working on the study ward, who completed an anonymous questionnaire. It involved no patients and collected no patient-identifiable or other sensitive data. The study was registered on the UHL NHS Trust Audit and Quality Improvement Programme on February 19, 2024 (reference 13079). In accordance with Trust policy, formal research ethics committee approval was not required for a project registered on this programme. Participation was voluntary, and completion of the anonymous questionnaire was taken as consent to participate.

## Results

Participants

A total of 32 nursing staff completed the questionnaire before the intervention, compared with 22 afterwards. Before the intervention, respondents included 20 nurses (62.5%) and 12 healthcare support workers (37.5%), whereas the post-intervention respondents comprised 17 nurses (77.3%) and five healthcare support workers (22.7%). There was no statistically significant difference in the proportion of nurses between the pre- and post-intervention surveys (p = 0.372).

Primary outcomes

The primary outcomes are shown in Table 1. The proportion of staff reporting falls prevention training within the previous 12 months rose from seven of 32 (21.9%) before the intervention to 20 of 22 (90.9%) after it (p < 0.001). The proportion reporting routine completion of falls risk assessment rose from 23 of 32 (71.9%) to 21 of 22 (95.5%) (p = 0.036). The proportion of staff reporting the highest level of confidence in their understanding of the UHL Falls Prevention Policy rose from 13 of 32 (40.6%) to all 22 respondents (100.0%) (p < 0.001). Consistent documentation of falls risk assessments and interventions was already high before the intervention (30 of 32; 93.8%) and remained high afterwards (21 of 22; 95.5%) (p = 1.000). Prompt reporting of falls rose from 28 of 32 (87.5%) to 21 of 22 (95.5%), but this difference was not statistically significant (p = 0.638).

**Table 1:** Primary outcomes before and after the educational intervention Data comprise the number of respondents with a positive response, with percentages of all respondents in that survey in parentheses (pre-intervention, n = 32; post-intervention, n = 22). The questionnaire number for each outcome is given in parentheses. On rating-scale questions, only a response of 5 was counted as positive. P-values were calculated using the two-tailed Fisher's exact test

Outcome measure (questionnaire number)	Pre-intervention survey, n (%), n = 32	Post-intervention survey, n (%), n = 22	P-value
Falls prevention training within the previous 12 months (Q4)	7 (21.9)	20 (90.9)	< 0.001
Routine completion of falls risk assessment (Q8)	23 (71.9)	21 (95.5)	0.036
Consistent documentation of falls risk assessments and interventions (Q10)	30 (93.8)	21 (95.5)	1.000
Prompt reporting of falls (Q14)	28 (87.5)	21 (95.5)	0.638
Highest level of confidence in understanding the policy (Q20)	13 (40.6)	22 (100.0)	< 0.001

Secondary outcomes

The secondary outcomes are presented in Table 2. Familiarity with the UHL Falls Prevention Policy increased from 21 of 32 respondents (65.6%) before the intervention to 21 of 22 (95.5%) afterwards (p = 0.017). Similarly, the proportion of staff reporting that they encouraged and assisted patients to use appropriate mobility aids increased from 21 of 32 (65.6%) to 21 of 22 (95.5%) (p = 0.017). Completion of falls risk assessments at the key points specified in the policy, namely on admission, following a fall and on transfer to a new ward, increased from 24 of 32 respondents (75.0%) to 21 of 22 (95.5%) (p = 0.067). Likewise, completion of core nursing and bedrail risk assessments for patients identified as being at high risk increased from 25 of 32 (78.1%) to 21 of 22 (95.5%) (p = 0.122). However, neither of these latter two differences reached statistical significance.

**Table 2:** Secondary outcomes before and after the educational intervention Data comprise the number of respondents with a positive response, with percentages of all respondents in that survey in parentheses (pre-intervention, n = 32; post-intervention, n = 22). The questionnaire number for each outcome is given in parentheses. On rating-scale questions, only a response of 5 was counted as positive. P-values were calculated using the two-tailed Fisher's exact test

Outcome measure (questionnaire number)	Pre-intervention survey, n (%), n = 32	Post-intervention survey, n (%), n = 22	P-value
Familiarity with the falls prevention policy (Q3)	21 (65.6)	21 (95.5)	0.017
Risk assessment on admission, after a fall and on ward transfer (Q9)	24 (75.0)	21 (95.5)	0.067
Core nursing and bedrail risk assessments for high-risk patients (Q11)	25 (78.1)	21 (95.5)	0.122
Encouraging and assisting use of appropriate mobility aids (Q15)	21 (65.6)	21 (95.5)	0.017

## Discussion

Principal findings

This before-and-after study found that a simple programme of repeated, ward-based small-group teaching was associated with substantial improvements in nursing staff training coverage, self-reported routine falls risk assessment, and confidence in understanding the UHL Falls Prevention Policy on a general medical ward. The most notable improvements were observed in training coverage, which increased from approximately one in five staff members to more than nine in 10, and confidence, with all respondents reporting the highest level of confidence following the intervention. Familiarity with the policy and the encouragement of appropriate mobility aid use also improved significantly, while completion of falls risk assessments at the specified time points and core nursing and bedrail assessments improved without reaching statistical significance. Documentation and falls reporting were already close to the departmental standard at baseline and showed no statistically significant changes. These findings suggest that the main gaps identified before the intervention related to access to training, falls risk assessment practices and staff confidence, rather than to reporting culture.

Comparison with existing literature

These findings are consistent with existing evidence that education is among the most effective strategies for preventing hospital falls [5] and that staff training can improve the adoption of recommended practices by healthcare professionals [6]. The intervention also incorporated several features associated with successful hospital falls prevention programmes. An intervention component analysis of 45 randomised trials found that integration within the local setting, particularly through the engagement of frontline ward staff, was present in most successful trials, although this alone was insufficient to reduce falls [10]. Similarly, qualitative research involving different stakeholder groups in acute hospital settings has highlighted the importance of adapting falls prevention programmes to the practical demands of ward workload and organisation [11]. Delivering teaching on the ward, in small groups and during routine shifts, provides a practical means of achieving this local integration while addressing the time and workload barriers frequently identified by clinicians [8,9].

The improvement in self-reported routine falls risk assessment is particularly relevant because current guidelines emphasise multifactorial assessment, rather than reliance on a risk-prediction score alone, as a central component of inpatient falls prevention [1,4]. Furthermore, the quality of existing clinical practice guidelines has been questioned due to variations in their methodological quality and inconsistencies in their recommendations [12]. Given that falls rates are among the highest in medical specialties [2], improving the consistency of falls risk assessment on medical wards represents an important target. The increase in staff confidence is also encouraging, as previous studies evaluating educational interventions for carers and nursing staff have reported corresponding improvements in knowledge, confidence and falls prevention behaviours [13]. However, confidence should not be equated with competence. The 100% highest-confidence rating observed after the intervention should therefore be interpreted cautiously, as high self-reported confidence may not necessarily reflect actual clinical competence or consistent adherence to recommended practices. Such gaps may only become apparent through direct observation or review of clinical documentation.

The present intervention primarily focused on staff education. However, recent evidence suggests that the impact of staff education on falls prevention may be greater when combined with patient and family involvement and interventions tailored to individual patients' needs and abilities [10,14]. Trials evaluating multicomponent care bundles and ward-based programmes for older inpatients have reported mixed effects on falls and hospital-associated complications [15,16]. This suggests that improving staff training and awareness, although important, should be considered an initial step rather than a standalone solution to inpatient falls prevention. Addressing the global challenge of falls requires coordinated efforts across the entire care pathway, supported by organisational leadership, a positive patient safety culture and sustained implementation strategies [17-19].

Implications for practice

This study suggests that improvements in training coverage, familiarity with the policy, staff confidence and self-reported practice can be achieved on a busy acute medical ward through teaching delivered during routine clinical shifts, without requiring additional funding or equipment. The approach may be transferable to other wards and hospitals, although its wider applicability was not evaluated and is likely to depend on local staffing levels, workload and leadership support. Evidence that improvements in healthcare professionals' practices often decline within six months of implementation [6] suggests that one-off teaching sessions are unlikely to sustain these improvements. We therefore recommend that ward-based falls prevention teaching be incorporated into the induction programme for new staff and reinforced through regular refresher sessions. Falls prevention practices should also be reassessed periodically as part of routine ward governance, and local falls prevention champions should be identified to support the ongoing implementation and sustainability of the programme [8,9].

Limitations

This study has several limitations. It was conducted on a single ward of one acute hospital, with small samples and no control group. This limits statistical power, generalisability to other settings (including larger hospitals), and the ability to attribute changes solely to the intervention. Fewer staff responded after the intervention (22) than before it (32). Because participation was voluntary, the two samples may differ in composition and engagement, which introduces possible selection and non-response bias. Because questionnaires were anonymous, responses could not be linked between surveys, and changes in ward staffing between surveys may have influenced the results. All analysed outcomes were self-reported and may be affected by social desirability bias, particularly after staff had received teaching on the expected answers. Knowledge and practice were not measured objectively.

The absence of significant changes in documentation and reporting may partly reflect ceiling effects and limited statistical power. Only the highest rating was counted as routine practice. This is a conservative threshold, but it may underestimate practice among staff who rated themselves at four. The questionnaire was developed locally from the policy and has not been formally validated. Response rates were not recorded. Several comparisons were made without adjustment for multiple testing, so individual p-values should be interpreted with caution. Finally, patient outcomes such as fall rates and falls-related injuries were not measured, so it is not possible to conclude that the intervention reduced falls. This limitation is shared by much of the implementation literature, where improved staff adoption of guideline recommendations has not consistently translated into fewer falls [6].

Future directions

Future studies should include objective measures of knowledge and practice, such as a validated knowledge test and a review of patient records to check completion of the multifactorial assessment and bedrail assessment. They should also measure patient outcomes, including falls per 1,000 occupied bed days and falls resulting in harm. Extending the programme to other medical wards, adding patient and family education, adopting a controlled design, and assessing whether improvements are sustained over longer periods would strengthen the evidence for this approach.

## Conclusions

A low-cost programme of repeated, ward-based small-group teaching delivered during routine clinical shifts was associated with improvements in nursing staff training coverage, familiarity with the hospital’s falls prevention policy, confidence in understanding it, and self-reported falls risk assessment practice on a busy general medical ward. The programme was feasible to deliver, required no additional funding or equipment, and could be adapted for other acute hospital settings. The findings should be interpreted with caution, because these outcomes were self-reported and the gains from staff education are known to fade without reinforcement. Regular ward-based education with periodic reassessment should therefore be embedded within ward governance structures. Future work should use objective measures of knowledge and practice, and should evaluate whether such improvements translate into fewer inpatient falls and less falls-related harm.

## Appendices

Falls Prevention Policy compliance questionnaire for nurses

The questionnaire was administered electronically using Google Forms. Questions are numbered from one to 21 in the order in which they appeared. Questions marked as required had to be answered before submission.

Section 1: General Information

Question 1. Please choose the title that best describes your role. Options: full-time nursing staff; agency or bank nursing staff; healthcare support worker. Single choice, required.

Question 2. Years of experience. Free text, required.

Section 2: Knowledge and Awareness

Question 3. Are you familiar with the hospital’s fall prevention policies? Options: yes; no. Required.

Question 4. Have you received training on fall prevention within the last year? Options: yes; no. Required.

Question 5. Which of the following is the most common risk factor for patient falls? Options: recent surgery; history of a fall in the previous year; delirium or dementia; use of drugs or alcohol; frailty; medical causes; other. Single choice, required.

Question 6. Which of the following is not a key principle of falls safety? Options: screening patients; identifying falls risks; reducing or managing falls risks; care planning; monitoring number of falls; communication. Single choice, required.

Question 7. Enhanced falls reduction measures will be commenced for all patients who (select all that apply): are aged 65 or over; have been admitted following a fall; are aged under 65 and assessed as at risk of falls using clinical judgement; have had two or more falls in the last 12 months; have had an inpatient fall in UHL; have had a mechanical fall. Required.

Section 3: Implementation of Fall Prevention Practices

Question 8. How often do you conduct fall risk assessments for patients? Five-point scale from one (never) to five (always). Required.

Question 9. When would fall risk assessments be performed for patients admitted at Leicester Royal Infirmary (select all that apply)? Options: at admission in the emergency department; in the assessment unit; when moved to a new ward; when they have a fall; weekly. Required.

Question 10. Do you consistently document fall risk assessments and interventions in the patient’s record? Options: yes; no. Required.

Question 11. When a patient is identified as high risk for falls, please select the interventions you implement (select all that apply): core nursing assessment for mobility; core nursing assessment for elimination; patient handling risk assessment; adult bedrail risk assessment. Required.

Question 12. What level of supervision do patients require after a fall? Options: one-to-one care; bay nursing. Single choice.

Question 13. When do you communicate fall risk information to other healthcare team members and relevant staff? Options: documenting screening for fall risk on Nervecentre immediately on arrival; within six hours of arrival; within 12 hours of arrival. Single choice, required.

Question 14. How often do you report a patient fall incident promptly following hospital policies? Five-point scale from one (never) to five (always). Required.

Section 4: Equipment and Environment

Question 15. How likely are you to encourage and assist patients in using appropriate assistive devices, such as walkers and handrails? Five-point scale from one (never) to five (always). Required.

Question 16. How likely are you to check the baseline mobility and equipment requirements of the patients? Five-point scale from one (never) to five (always). Required.

Question 17. Which of the following do you encourage in patients to reduce the risk of falls (select all that apply)? Options: switching to decaffeinated drinks; having the last drink two hours before bedtime; correct measurement of lying and standing blood pressure; keeping the call bell within reach; accompanying patients inside the bathroom.

Section 5: Continuous Improvement and Feedback

Question 18. Do you feel there is a system in place for nurses to provide feedback on fall prevention policies and practices? Options: yes; no. Required.

Question 19. How do you stay updated on the latest fall prevention strategies and best practices (select all that apply)? Options: reviewing UHL falls prevention guidelines; falls prevention teaching sessions; training programmes; other. Required.

Question 20. How would you rate your overall confidence regarding your understanding of UHL fall prevention policies? Five-point scale from one (low) to five (high). Required.

Question 21. Comments and additional information. Free text

## References

[1] Montero-Odasso M, van der Velde N, Martin FC (2022). World guidelines for falls prevention and management for older adults: a global initiative. Age Ageing.

[2] Heikkilä A, Lehtonen L, Junttila K (2023). Fall rates by specialties and risk factors for falls in acute hospital: a retrospective study. J Clin Nurs.

[3] Dykes PC, Curtin-Bowen M, Lipsitz S (2023). Cost of inpatient falls and cost-benefit analysis of implementation of an evidence-based fall prevention program. JAMA Health Forum.

[4] Vandervelde S, Skelton DA, Milisen K, Treml J, Martin FC (2025). Commentary on NICE guidance 249-falls: assessment and prevention in older people and in people 50 and over at higher risk. Age Ageing.

[5] Morris ME, Webster K, Jones C (2022). Interventions to reduce falls in hospitals: a systematic review and meta-analysis. Age Ageing.

[6] Haley MN, Sherrington C, Lawler K, Harding KE, Lord M, Williams S, Taylor NF (2025). Falling short on implementation of fall prevention guidelines in health services: a systematic review with meta-analysis. Age Ageing.

[7] Seppala LJ, Lord SR, Masud T, Montero-Odasso M, Ryg J, Tan MP, van der Velde N (2025). The world falls guidelines: how is implementation progressing globally?. Age Ageing.

[8] Heng H, Kiegaldie D, Slade SC (2022). Healthcare professional perspectives on barriers and enablers to falls prevention education: a qualitative study. PLoS One.

[9] Shaw L, Kiegaldie D, Heng H, Morris ME (2023). Interprofessional education to implement patient falls education in hospitals: lessons learned. Nurs Open.

[10] McLennan C, Sherrington C, Suen J (2024). Features of effective hospital fall prevention trials: an intervention component analysis. BMC Geriatr.

[11] McLennan C, Sherrington C, Tilden W (2024). Considerations across multiple stakeholder groups when implementing fall prevention programs in the acute hospital setting: a qualitative study. Age Ageing.

[12] Montero-Odasso MM, Kamkar N, Pieruccini-Faria F (2021). Evaluation of clinical practice guidelines on fall prevention and management for older adults: a systematic review. JAMA Netw Open.

[13] Wang Q, Zou H, Wang Q (2022). The effectiveness of multimedia combined with teach-back method on the level of knowledge, confidence and behavior of professional caregivers in preventing falls in elderly patients: a randomized non-blind controlled clinical study. Medicine (Baltimore).

[14] Morris ME, Thwaites C, Lui R (2024). Preventing hospital falls: feasibility of care workforce redesign to optimise patient falls education. Age Ageing.

[15] Di Gennaro G, Chamitava L, Pertile P (2024). A stepped-wedge randomised controlled trial to assess efficacy and cost-effectiveness of a care-bundle to prevent falls in older hospitalised patients. Age Ageing.

[16] Mudge AM, McRae P, Banks M (2022). Effect of a ward-based program on hospital-associated complications and length of stay for older inpatients: the cluster randomized CHERISH trial. JAMA Intern Med.

[17] Seppala LJ, van der Velde N (2022). How to tackle the global challenge of falls?. Age Ageing.

[18] Close JC, Lord SR (2022). Fall prevention in older people: past, present and future. Age Ageing.

[19] Mistri IU, Badge A, Shahu S (2023). Enhancing patient safety culture in hospitals. Cureus.

